# Efficient and Precise Micro-Injection Molding of Micro-Structured Polymer Parts Using Micro-Machined Mold Core by WEDM

**DOI:** 10.3390/polym11101591

**Published:** 2019-09-29

**Authors:** Qianghua Liao, Chaolan Zhou, Yanjun Lu, Xiaoyu Wu, Fumin Chen, Yan Lou

**Affiliations:** 1 School of Mechanical and Electrical Engineering, Shenzhen Polytechnic, Shenzhen 518055, China; liaoqianghua@szpt.edu.cn; 2 Guangdong Provincial Key Laboratory of Micro/Nano Optomechatronics Engineering, College of Mechatronics and Control Engineering, Shenzhen University, Shenzhen 518060, China; wuxy@szu.edu.cn (X.W.); 2172291706@email.szu.edu.cn (F.C.); susanlou121@163.com (Y.L.)

**Keywords:** micro-structure, polymer, micro-injection molding, mold, WEDM

## Abstract

In this paper, micro-structured polymer parts were efficiently and accurately fabricated by micro-injection molding using a micro-structured mold core machined by wire electrical discharge machining (WEDM). The objective was to realize low-cost mass production and manufacturing of micro-structured polymer products. The regular micro-structured mold core was manufactured by precise WEDM. The micro-structured polymer workpieces were rapidly fabricated by micro-injection molding and the effects of the micro-injection molding process parameters on replication rate and surface roughness of micro-structured polymers were systematically investigated and analyzed. It is shown that the micro-structured polymer can be rapidly and precisely fabricated by the proposed method. The experimental results show the minimum size machining error of the micro-structured mold core and the maximum replication rate of micro-formed polymer were 0.394% and 99.12%, respectively. Meanwhile, the optimal micro-injection molding parameters, namely, jet temperature, melt temperature, injection velocity, holding pressure and holding time were 195 °C, 210 °C, 40 mm/min, 7 Mpa and 5 s, respectively. The surface roughness *R*_a_ at the groove bottom and top of the micro-structured polymer workpieces achieved minimum values of 0.805 µm and 0.972 µm, respectively.

## 1. Introduction

Surfaces with micro/nano features on polymer components have been widely used in optical displays, microfluidic chips, cell and tissue cultures, and other roles [1,2,3,4]. For example, a distribution expression of microprism was designed on the bottom surface of an integrated light guide plate (ILGP). The simulation results showed that the luminance uniformities of the integrated backlight modules (BLMs) were higher than 85% [1]. A microfluidic chip combining poly-DL-lactide (PLA) patterned electrospun fibers with polydimethylsiloxane (PDMS) was successfully developed by lithography to realize toxicity screening of nanoparticles [2]. A 3D artificial polymeric scaffold with sub-micrometer spatial resolution was fabricated to promote cell growth and tissue expansion [3]. A lab-on-a-chip (LoC) with polymer substrate was fabricated for the application of non-invasive and contactless mechanical phenotyping of single cancer cells [4].

Due to its simple manufacturing process, high forming quality and low production cost, micro-injection molding is suitable for mass production and manufacturing of micro-structured polymers, and it is almost unlimited in the geometric shape of plastic parts. Therefore, it has become the main molding process of micro-structured polymer [5,6,7]. For instance, the disposable optical stretcher and microfluidic devices can be rapidly and precisely fabricated by micro-injection molding [8,9]. The micro features with a width of 5 µm and a depth of 15 µm were precisely replicated by micro injection molding [10]. The square pillared array structures with the sizes of 40 nm in width and 107 nm in height (aspect ratio > 2.5) were fabricated by micro injection molding [11]. The micro-structured surfaces with high aspect ratio can be also successfully replicated by micro injection molding [12]. The feasibility and availability of the micro injection molding technique had been demonstrated in the presented works. The forming quality and replication accuracy of micro-injection molded polymer products mainly depend on the machining quality and accuracy of the micro-structured mold core. The common micro-machining technologies of the micro-structured mold core include electrochemical etching [13], laser machining [14] and micro cutting machining [15]. However, it is difficult to simultaneously ensure the macroscopic shape accuracy and the surface quality of 3D micro-structures at the micron scale.

It is known that wire electrical discharge machining (WEDM) [16,17,18] is suitable for machining complicated micro-structures with micron sizes. For instance, micro-grooved structures with size less than 100 μm were fabricated on the surface of Ti_3_SiC_2_ ceramic by WEDM to improve hydrophobic performance [17]. A micro ball-ended probe with the diameter of 97.6 μm and surface roughness *R*_a_ of 0.7 μm was successfully fabricated by micro-WEDM [18]. However, it is relatively difficult to machine regular microarray structures with high 3D microscopic shape accuracy. In order to obtain high surface quality and shape accuracy, micro-structures with a size within 100 microns are only processed by low-speed WEDM. Therefore, high-precision low-speed WEDM was proposed to fabricate micro-structures on the surface of the mold core to realize efficient mass-production and manufacturing of micro-structured polymer parts by micro-injection molding.

In addition to the machining quality and precision of the micro-structured mold core, the micro-injection molding process parameters also affect the forming quality of micro-structured products. Generally, micro-injection molding has five steps, namely, injecting, packing, holding, cooling and demolding. Due to the tiny structural size and volume of micro-injection molded parts, it is difficult to ensure the shape accuracy and surface quality of injection molded micro-structured parts under macroscopic injection molding process conditions [19,20]. Therefore, in this work, the influences of micro-injection molding process parameters on the micro-forming accuracy and surface quality of a micro-structured polymer workpiece were systematically investigated.

In this paper, the efficient and precise WEDM was firstly used to machine rectangular microarray structures on the surface of mold core. Then, the micro-injection molding technology was used to rapidly fabricate micro-structured polymer parts. Next, the influences of injection molding process parameters, such as jet temperature, melt temperature, injection velocity, holding pressure and holding time on the shape accuracy and replication rate of micro-structured polymer were analyzed. Finally, the process conditions of micro-injection molding were optimized.

## 2. Materials and Methods

### 2.1. Wire Electrical Discharge Machining (WEDM) of Mold Core with Micro-Structures

**Figure 1:**
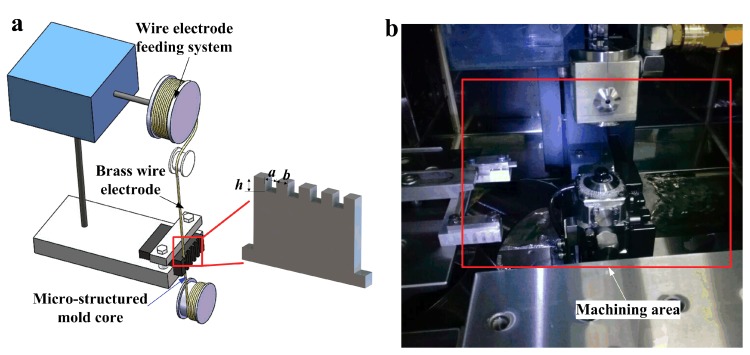
The processing schematic diagram of mold core machined by wire electrical discharge machining (WEDM): (**a**) schematic diagram of WEDM; (**b**) machining scene.

As a promising mold material, titanium silicon carbide (Ti_3_SiC_2_) combines the properties of both metal and ceramic, and has high strength, fracture toughness, good thermal stability and workability, and good self-lubrication and demolding performance [21,22]. Commercial Ti_3_SiC_2_ bulk was cut into a rectangular block with a size of 15 mm × 15 mm × 5 mm and mounted on the worktable of a wire electrical discharge machining machine (AP250LS, Sodick, Yokohama, Japan). The conductive Ti_3_SiC_2_ mold core was regarded as the cathode and was immersed into dielectric oil (Glysantin G 48–24, BASF, Ludwigshafen, Germany) (see Figure 1). An anodic brass wire electrode with diameter of 50 μm was employed to fabricate regular micro-structures on the surface of the Ti_3_SiC_2_ mold core. Figure 1 shows the processing schematic diagram and machining scene of the mold core using WEDM. The designed micro-grooved structure parameters, namely, groove width *a*, groove depth *h* and groove spacing *b*, were all set as 100 μm (see Figure 1a). Gradually, the rectangular micro-array structures with macroscopic length and thickness of 7 mm × 1.6 mm were successfully fabricated on the surface of the mold by WEDM under the driving of the wire electrode feeding system along a predesigned machining path. Finally, the machined micro-structured mold core was cleaned by alcohol three times in an ultrasonic cleaning machine.

### 2.2. Micro-Injection Molding of Polymer Workpieces with Micro-Structures

**Figure 2:**
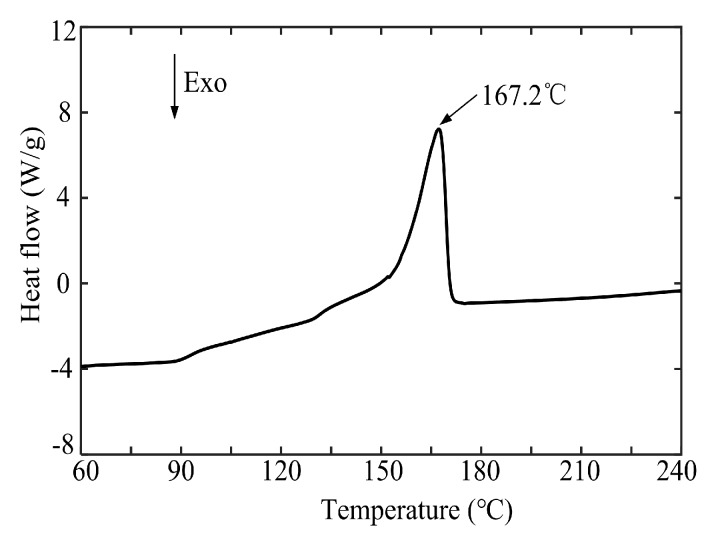
The differential scanning calorimetry (DSC) heating curve of the polymer particles.

Polypropylene (PP) particles (B310, Lotte Chemical Corporation, Seoul, Korea) were employed as the polymer workpiece material. The density of the polymer particle material was 0.9 g/cm^3^. Its theoretical thermal deformation temperature and melting point were 110 °C and 167 °C, respectively. Before micro-injection molding, differential scanning calorimetry (DSC 8000, PerkinElmer, Wellesley, MA, USA) testing of polymer particles was conducted. Figure 2 shows the DSC heating curve of the polymer particles. During the heating process in DSC, one sharp endothermic peak appeared at nearly 167.2 °C, which was close to the melting point of the polymer. Therefore, in this work, the jet temperature and melt temperature should be set at a temperature higher than 167.2 °C to guarantee complete melting of polymer particles before injection into the mold core.

**Figure 3:**
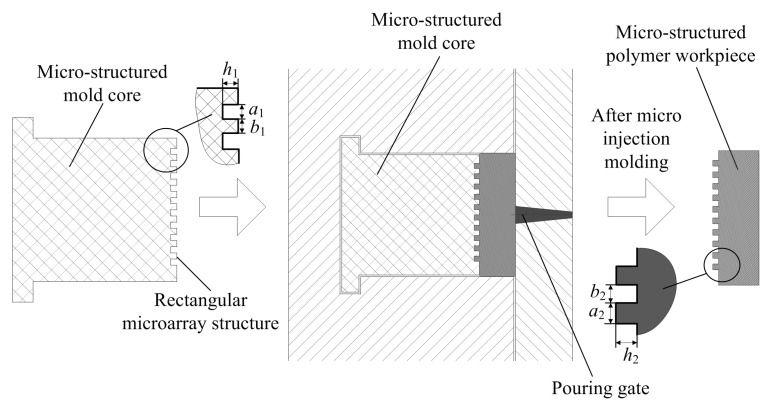
The schematic diagram of micro-injection molding of micro-structured polymer.

Micro-structured polymer components can be rapidly fabricated by micro-injection molding technology using the micro-structured mold core machined by WEDM. Figure 3 shows the principle and forming process of micro-injection molding of micro-structured polymer. The polymer particles were put into a micro-injection molding machine (Babyplast 6/10P, Cronoplast Sl, Barcelona, Spain). The micro-injection molding machine has the advantages of small size, high molding precision and high efficiency, and thus is more suitable for mass production and manufacturing of small precision polymer parts. As seen in Figure 3, in the whole micro-injection molding process, the polymer particles were first heated and plasticized, then injected into the mold core through the pouring gate. After pressure holding, cooling and demolding, the rectangular microarray structures on the surface of the micro-structured mold core can be replicated on the surface of the polymer workpiece. Therefore, the micro-structured polymer workpieces were rapidly fabricated by micro-injection molding.

We further investigated the effects of micro-injection molding process parameters on the micro-forming quality of molded polymer workpieces. According to previous experiments, when the mold temperature was set at room temperature, the micro-structured polymer samples can be well-formed [6], so the mold temperature was not considered as an injection molding process parameter in this paper. In order to simplify test times, five common process parameters, namely, jet temperature (the heating temperature of nozzle), melt temperature (the temperature of the melt polymer entering the cavity), injection velocity, holding pressure and holding time, were selected and set according to the single factor testing method in the experiments. The micro-injection molding parameters and corresponding levels in designed experiments are listed in Table 1. Because one set of process parameters were repeated four times, there were in total 21 sets of micro-structured polymer. Samples corresponded to the 21 sets of micro-injection molding process parameters shown in Table 1. Under each process parameter, ten micro-injection molded workpieces were selected for testing in order to reduce the experiment error as much as possible.

### 2.3. Measurement of Micro-Structured Mold Core and Polymer Workpieces

The Raman spectrum (LabRAM HR Evolution, Horiba Jobin Yvon, France) was employed to detect the shift in phase constitutions of the mold core before and after WEDM processing. The surface topographies of micro-machined mold cores and micro-formed polymer workpieces were characterized by high-resolution scanning electron microscope (SEM, Apreo S, FEI Company, Hillsboro, OR, USA). The 3D topographies and section profile curves of micro-structured mold cores and polymer workpieces were captured by non-contact 3D laser scanning microscope (VK-250, Keyence, Osaka, Japan). Since the top and bottom of the workpiece and mold core were not absolutely horizontal, the surface roughness of *R*_a_ was uniformly used to evaluate the surface qualities of machined mold core and formed polymer workpieces. Through the measured micro-grooved profile curves, the groove width, groove depth and groove spacing of mold cores and polymer surfaces could be obtained. The presented experimental results including the micro-groove size parameters and surface roughness *R*_a_ were the average values of five measurements.

## 3. Results and Discussions

### 3.1. Phase Constitutions of the Micro-Structured Mold Core Surfaces

**Figure 4:**
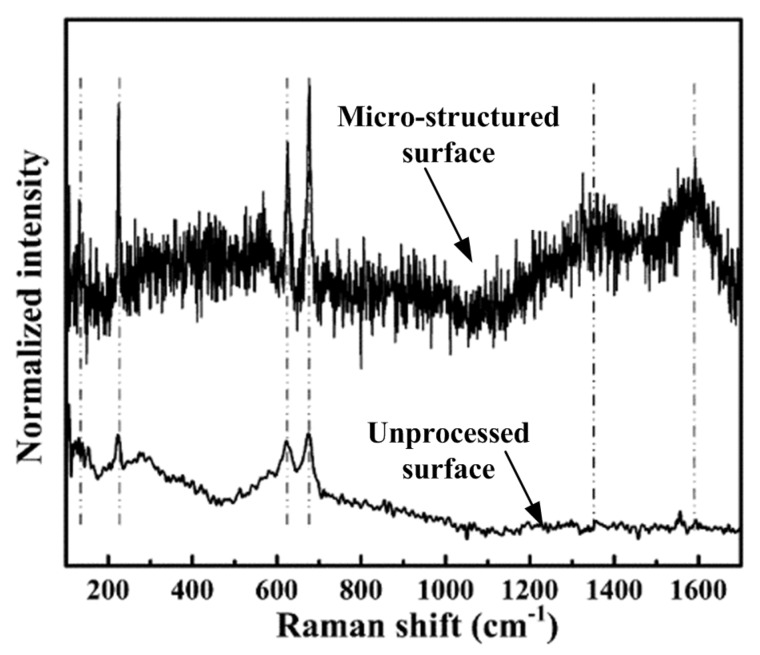
The Raman spectra of the unprocessed and micro-structured Ti_3_SiC_2_ mold core surface.

Figure 4 shows the Raman spectra of the unprocessed and micro-structured Ti_3_SiC_2_ mold core surface. The spectra of the unprocessed surface had three sharp peaks at 223 cm^−1^, 623 cm^−1^ and 675 cm^−1^, and one broad peak at 276 cm^−1^, which correctly corresponded to the E2g, A1g, E1g, and A1g modes of Ti_3_SiC_2_, respectively [23], indicating that the major phase on the surface was Ti_3_SiC_2_. Compared with the unprocessed surface, the micro-structured surface exhibited a wavier spectrum, especially for the vibrational modes ranging from 450 cm^−1^ to 750 cm^−1^, which indicated the existence of titanium carbide [24]. The vanishing peak at 276 cm^−1^, accompanied with the appearance of two broad peaks at 1365 cm^−1^ and 1586 cm^−1^, reflected the decreasing content in Ti_3_SiC_2_ and increasing disordered carbon [25]. Compared with the peaks assigned to Ti_3_SiC_2_, the peaks assigned to the impurity were relatively lower than the former, indicating that the decomposition of Ti_3_SiC_2_ on the micro-structured surface was limited. Therefore, the chemical composition of the Ti_3_SiC_2_ mold core surface was hardly affected by electrical discharge machining.

### 3.2. Topographies and Section Profile Curves of Micro-Structured Mold Core Surfaces

**Figure 5:**
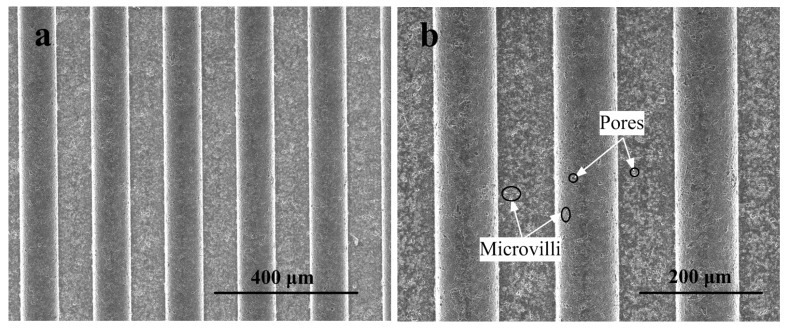
SEM photos of the micro-structured mold core surface: (**a**) surface topography of machined mold core; (**b**) the magnified version of Figure 5a.

Figure 5 shows the SEM photos of the micro-structured mold core surface. As seen from Figure 5, the regular micro-structured structures were successfully machined on the surface of the mold core by WEDM. Figure 5b is an amplified version of Figure 5a. As shown in Figure 5b, a certain number of small pores and microvilli produced by the decomposition of Ti_3_SiC_2_ into TiC and disordered carbon [24] were distributed on the groove top and bottom, which were in accordance with the results of the Raman spectrum. Compared with the groove top, the bottom had a more uniform distribution of pores and microvilli because electrical erosion mainly occurred in the edge regions of the groove top during the machining process.

**Figure 6:**
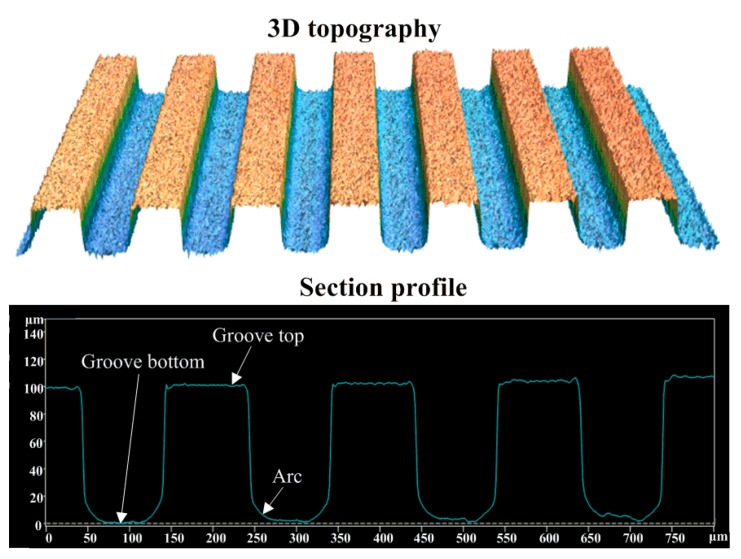
3D topographies and section profile curves of micro-structured mold core surface.

Figure 6 shows the 3D topographies and section profile curves of the micro-structured mold core surface. Table 2 shows the surface roughness *R*_a_ and groove size parameters of the designed and machined micro-structured mold cores. As seen from Table 2, the micro-machined groove width *a*_1_, groove depth *h*_1_ and groove spacing *b*_1_ were almost in accordance with the designed micro-groove sizes. The machined width error, depth error and spacing error were 0.317 µm, 0.399 µm and 0.465 µm, respectively. The machined arc curve at the groove bottom side can be observed on the contour of the micro grooves, and was mainly attributed to the cycloidal cross-section of the brass electrode, leading to the existence of machining error. The surface roughness *R*_a_ of the groove top and bottom of the micro-structured mold core machined by WEDM were 1.354 µm and 1.476 µm, respectively.

According to the measured section profile curves before and after WEDM, the relative machining errors of the micro-structured mold core, namely, relative width error *Δa*_m_, relative depth error *Δh*_m_ and relative spacing error *Δb*_m_ can be obtained by the following three equations:

*Δa*_m_ = (|*a* − *a*_1_|/*a*) × 100% (1)

*Δb*_m_ = (|*b* − *b*_1_|/*b*) × 100% (2)

*Δh*_m_ = (|*h* − *h*_1_|/*h*) × 100% (3)

By calculating the average value of the three relative machining errors, the average size machining error of WEDM *ε*_m_ can be obtained by the following formula:

*ε_m_* = (*Δa*_m_ + *Δb*_m_ + *Δh*_m_)/3 (4)

After calculation by the above equations, the size machining error *ε*_m_ of the micro-structured mold core using WEDM was as low as 0.394%. Therefore, the regular micro-structured mold core can be precisely machined by WEDM, which was beneficial to produce injection molded polymer parts with high forming quality.

### 3.3. Topographies and Section Profile Curves of Micro-Structured Polymer Surfaces

**Figure 7:**
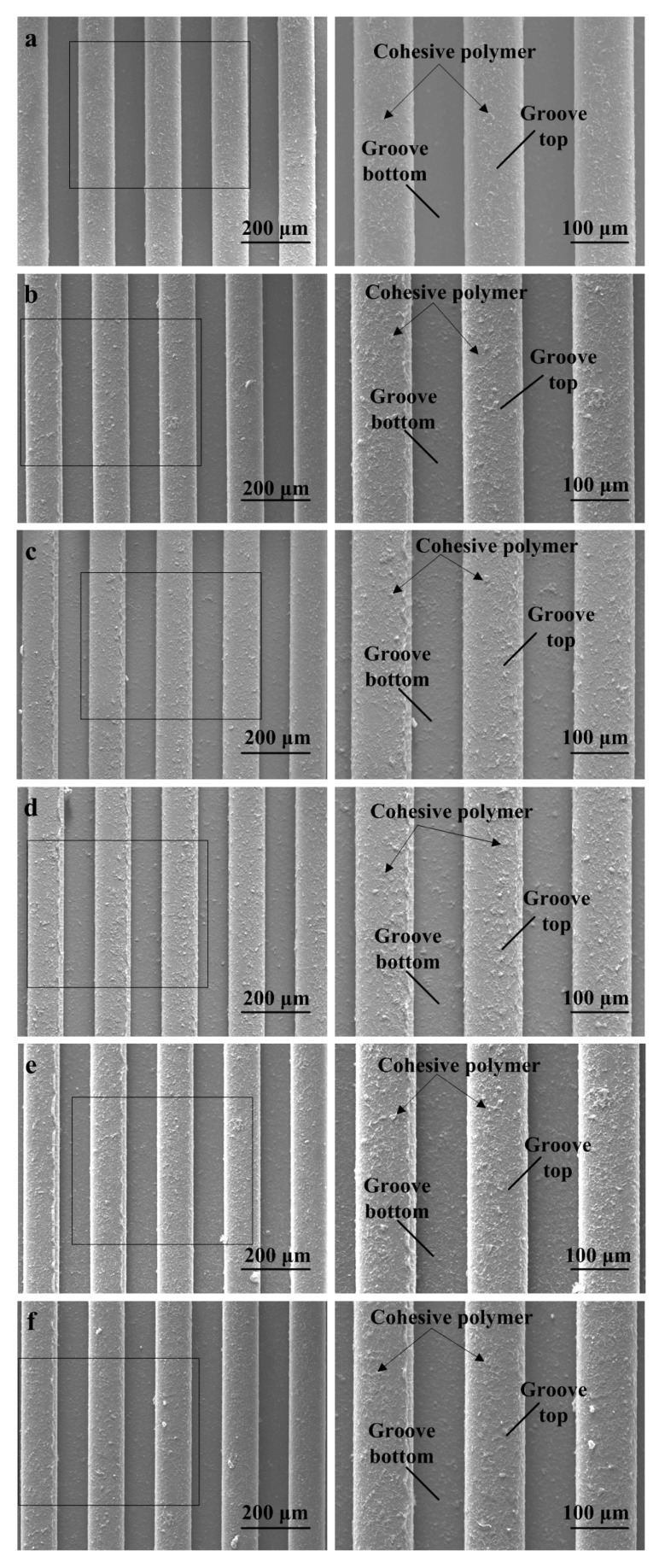
SEM photos of the selected micro-structured polymer samples: (**a**) Sample 3; (**b**) Sample 5; (**c**) Sample 6; (**d**) Sample 11; (**e**) Sample 17; (**f**) Sample 25.

After the micro-injection molding experiments in Section 2.2 were implemented, 21 sets of micro-structured polymer samples were obtained. In order to distinguish the molding quality under different injection molding process parameters, the sample with the best molding quality (experiment No. 3 in Table 1), as well as five groups of test samples with the worst molding quality corresponding to five process parameters were selected. The selected micro-formed polymer Samples 5, 6, 11, 17 and 25 were obtained when changing one process parameter and maintaining the other parameters, which corresponded to jet temperature, melt temperature, injection velocity, holding pressure or holding time, respectively. Figure 7 shows the SEM photos of the selected micro-injection molded polymer samples. It can be observed that the rectangular micro-array structures on the surface of the molded polymer samples were roughly in accordance with that of the machined mold core (see Figure 5). It is shown that micro-features on the surface of the micro-structured mold cores machined by WEDM were successfully replicated on the micro-formed polymer surfaces, which indicated that the molten polymer almost entirely filled into the micro-structures on the mold core surface. According to the SEM topographies shown in Figure 7, the surface of Sample 3 (see Figure 7a) had the least adhesive polymer debris compared with other samples (see Figure 7b–f). The SEM photo on the left is a larger version of the photo on the right for a sample. It is found that the rectangular groove bottom surface had better forming quality than the one of the groove top for all polymer samples. This is because the surface quality of the groove top and groove bottom of injection molded polymer workpieces mainly depends on the surface quality of the groove bottom and groove top of the micro-structured mold core, respectively. Because the groove top surface of the mold core had been polished in advance, the surface roughness *R*_a_ of the unprocessed groove top of the mold core was less than that of the groove bottom machined by WEDM (see Table 2). Compared with the groove bottom surface, the surface at the groove top of the micro-structured mold core was smoother, leading to smoother surfaces of polymer samples at groove bottom against groove top.

In addition, it is also seen that there was significant residual cohesive polymer debris on the surface of the groove top for all micro-formed polymer samples. This is because the polymer materials shrank and deformed after heating and cooling in the micro-injection molding process. Therefore, the micro-groove of the micro-structured mold core cannot be completely filled with molten polymers. Furthermore, it is found that the residual melted polymer debris on the groove top surface of Sample 3 (see Figure 7a) was the least compared to other samples (see Figure 7b–f). It is clearly observed that the size and number of cohesive polymers on the surface of different samples corresponding to different micro-injection molding process parameters were different (see Figure 7b–f). It is confirmed that the micro-forming quality of micro-structured polymer was related to the micro-injection molding process parameters. Therefore, it is necessary to choose appropriate micro-injection molding process parameters in the production and manufacturing of micro-structured polymer parts.

**Figure 8:**
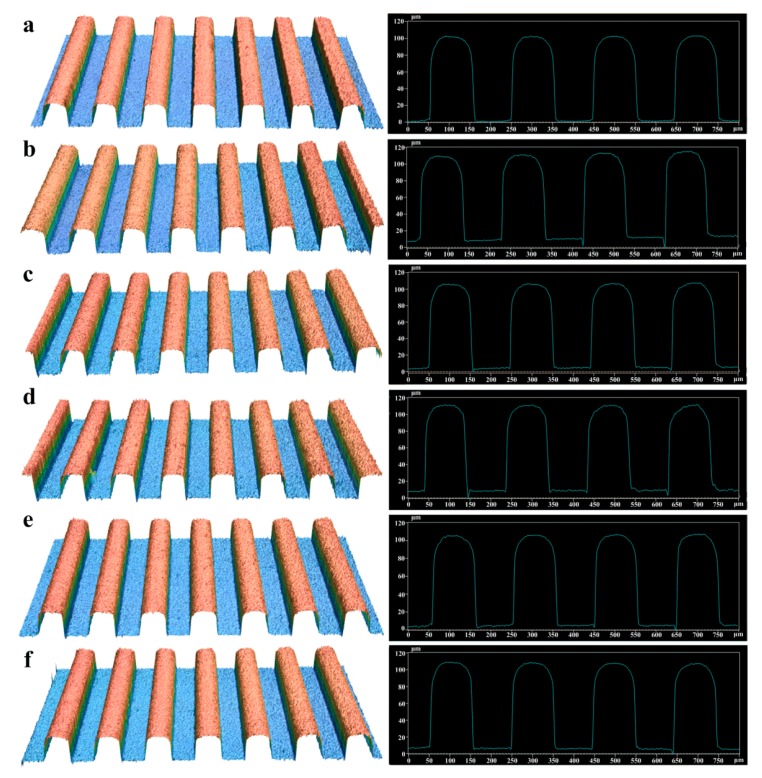
3D topographies of the micro-structured polymer samples: (**a**) Sample 3; (**b**) Sample 5; (**c**) Sample 6; (**d**) Sample 11; (**e**) Sample 17; (**f**) Sample 25.

Figure 8 shows the 3D topographies and section profile curves of selected micro-injection molded polymer samples. The selected samples shown in Figure 8 correspond to those shown in Figure 7. The results of 3D topographies and profiles shown in Figure 8 further confirm the SEM results shown in Figure 7. Compared with other samples, Sample 3 had a relatively smooth surface at groove tops and bottoms. Through the measured profile curves of rectangular micro-groove structures, the micro-structure size parameters of micro-injection molded polymer workpieces including groove width a_2_, groove depth h_2_ and groove spacing b_2_ can be acquired. The surface roughness *R_a_* of groove bottom and groove top on micro-formed polymer surfaces can be also obtained.

Table 3 shows the surface roughness and micro-structure size parameters of molded polymer workpieces. As seen from Table 3, the surface roughness *R*_a_ on the surface of the groove bottom was less than that on the surface of the groove top for all micro-structured polymer samples. This is because the surface quality at the groove bottom of micro-structured polymer mainly depends on the surface quality at the groove top of the mold core. Moreover, comparing the surface roughness *R*_a_ of the mold core shown in Table 2 with polymer workpieces shown in Table 3, it is found that the surface roughness of polymers was less than that of the mold core. The reason may be that the melt polymer did not fill all peaks and valleys due to trapped air, resulting in a better roughness. According to the results of Table 3, in the process of micro-injection molding, the average width error, average depth error and average spacing error were 1.277 µm, 1.158 µm and 1.050 µm, respectively. Because the designed original theoretical sizes of micro-structures were 100 µm, the micro-forming accuracy of micro-structured polymer was only about 1%. Moreover, under different micro-injection molding parameters, the groove size parameters and surface roughness of micro-structured polymer workpieces were different (see Table 3). Therefore, it is necessary to investigate the effects of micro-injection molding parameters on the micro-forming quality and replication precision of micro-structured polymer.

### 3.4. Effects of Micro-Injection Molding Parameters on Replication Rate of Micro-Structured Polymer

According to the measured section profile curves before and after micro-injection molding and the data results shown in Table 3, the relative micro-forming errors of micro-structured polymer, namely, relative width error *Δa*_w_, relative depth error *Δh*_w_ and relative spacing error *Δb*_w_ can be calculated as follows:

*Δa*_w_ = (|*b*_2_ − *a*_1_|/*a*_1_) × 100% (5)

*Δb*_w_ = (|*a*_2_ − *b*_1_|/*b*_1_) × 100% (6)

*Δh*_w_ = (|*h*_2_ − *h*_1_|/*h*_1_) × 100% (7)

The replication precision of micro-features on the mold core surface, which decided the forming quality of micro-injection molding, can be evaluated by the filling rate. In this work, the replication rate was the main output target to evaluate the replication quality of molded polymer workpieces. The replication rate was defined as the ratio of each micro-structural volume of micro-injection molded part to the micro-structural volume of the mold core. As mentioned in the previous experiment, the thickness of both micro-structured polymer and mold core was 1.6 mm. Therefore, the replication rate was only related to the average depth, width and spacing of the micro-structure sizes. The micro-forming accuracy of micro-injection molding *ε*_w_ can be computed by the following equation:

*ε_w_* = (*Δa*_w_ + *Δb*_w_ + *Δh*_w_)/3 (8)

Therefore, the replication rate of micro-injection molding *γ* can be obtained by the following formula:

*γ* = 100% − *ε*_w_ (9)

**Figure 9:**
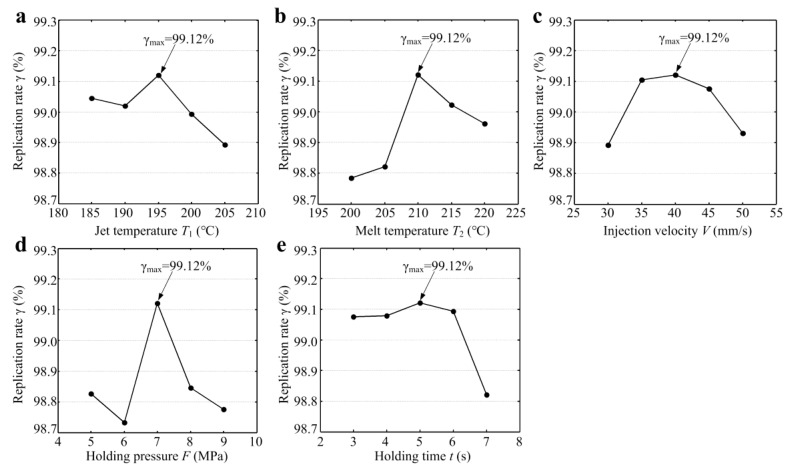
The effects of micro-injection molding parameters on replication rate *γ*: (**a**) jet temperature *T*_1_; (**b**) melt temperature *T*_2_; (**c**) injection velocity *V*; (**d**) holding pressure *F*; (**e**) holding time *t*.

Based on the above equations, the effects of micro-injection molding parameters on the replication rate of micro-structured polymers are depicted in Figure 9. As seen from Figure 9, when the micro-injection molding parameters, namely, jet temperature T_1_, melt temperature T_2_, injection velocity V, holding pressure F and holding time t were 195 °C, 210 °C, 40 mm/min, 7 Mpa and 5 s, respectively, the replication rate γ of micro-formed polymer reached a maximum value of 99.12%. There was no significant change in the replication rate when the jet temperature increased. The replication rate was ranged from 98.89% to 99.12% with the change of jet temperature. The replication rate achieved a maximum value of 99.12% until the jet temperature increased to 195 °C (see Figure 9a). With the continual increase of jet temperature, the replication rate rapidly decreased. The reason may be that too high jet temperature affects the flow rate and injection volume of melted polymer, leading to incomplete injection filling. Figure 9b shows the effect of melt temperature on the replication rate of polymer workpieces. The replication rate first dramatically increased, and then slowly decreased with the increase of melt temperature. The range of replication rate was 98.78% to 99.12% with the change of melt temperature. This indicated that the melt temperature cannot be too high or too low. It would produce uneven forming of melt polymer if the melt temperature was too low. A melt temperature that is too high would cause excess shrinkage in cooling and solidification. The effect of injection velocity on the replication rate is shown in Figure 9c. The replication rate first increased, and then rapidly decreased with the increase of injection velocity. The replication rate was ranged from 98.89% to 99.12% with the change of injection velocity. As the injection velocity ranged from 35 mm/s to 45 mm/s, the variation of the replication rate was insignificant. This indicated that the proper injection velocity was conducive to sufficient filling and replication of melted polymer in the process of micro-injection molding. The possible reasons are that it would affect the flow rate of melted polymer if the injection velocity was too slow, leading to incomplete filling for micro-structure features. If the injection velocity was too fast, it would cause uneven filling between melted polymer and the mold core. In Figure 9d, the highest replication rate only can be achieved under the holding pressure of 7 MPa, while a holding pressure higher or lower than 7 MPa would lead to a sharp decrease in the replication rate. This illustrated that the holding pressure had a large influence on micro-forming quality and replication rate. The replication rate was ranged from 98.74% to 99.12% with the change of holding pressure. Figure 9e shows the effect of the holding time on the replication rate of polymer workpieces. Although the optimal holding time was 5 s, the replication rate smoothly increased when the holding time ranged from 3 s to 6 s, and then prominently decreased when the holding time exceeded 6 s. The replication rate was ranged from 98.81% to 99.12% with the change of holding time. Compared with injection velocity and jet temperature, the effects of the melt temperature, holding pressure and holding time on the replication rate of micro-structured polymer were more significant in the process of micro-injection molding.

### 3.5. Effects of Micro-Injection Molding Parameters on Surface Quality of Micro-Structured Polymer

**Figure 10:**
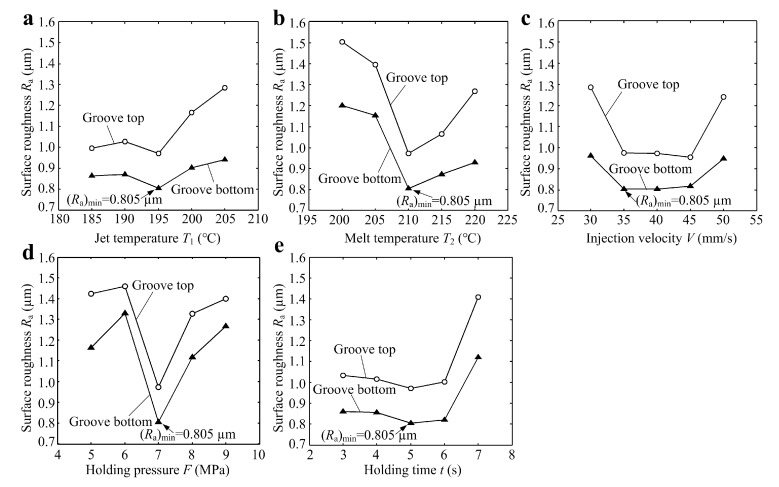
The effects of micro-injection molding parameters on surface roughness *R*_a_: (**a**) jet temperature *T*_1_; (**b**) melt temperature *T*_2_; (**c**) injection velocity *V*; (**d**) holding pressure *F*; (**e**) holding time *t*.

The surface roughness *R_a_* at the groove top and bottom of micro-structured polymers was obtained by measured section profiles (see Table 3). Figure 10 shows the effects of micro-injection molding parameters on surface roughness *R_a_*. It is shown that the surface roughness at the groove top was larger than that at the groove bottom under the same micro-injection molding parameters. The increasing or decreasing trends of surface roughness at the groove top and bottom with the changes of micro-injection molding parameters were consistent. As seen from Figure 10, when the micro-injection molding parameters, namely, jet temperature T_1_, melt temperature T_2_, injection velocity V, holding pressure F and holding time t were 195 °C, 210 °C, 40 mm/min, 7 Mpa and 5 s, respectively, the surface roughness *R_a_* at the groove bottom of the micro-structured polymer achieved a minimum value of 0.805 µm. Meanwhile, the replication rate reached the maximum value of 99.12% (see Figure 9).

The surface roughness at the groove top and bottom first slowly decreased, then dramatically increased with the increase of jet temperature (see Figure 10a). This is because a jet temperature that was too high was not conducive to the flow of molten polymer, leading to the rapid reduction in replication rate and increase in surface roughness. Figure 10b shows that the surface roughness first rapidly decreased, then increased with the increasing melt temperature. This indicated that there was a large influence of melt temperature on surface roughess. It is seen that the surface roughness first significantly decreased, then slowly increased with the increase of injection velocity (see Figure 10c). This is because an injection speed that was too slow or too fast would cause the melted polymer to not fully fill the micro-structured mold core. Figure 10d shows that the surface roughness first rapidly decreased, then sharply increased with the increase of holding pressure. This indicated that the holding pressure had a great influence on the surface roughness. It shows that the surface roughness first slowly decreased, then greatly increased with the increase of holding time (see Figure 10e). As a result, compared with injection velocity, jet temperature and holding time, the melt temperature and holding pressure had greater influence on the surface roughness of micro-formed polymer.

## 4. Conclusions

The highly efficient and precise wire electrical discharge machining (WEDM) technology is proposed to fabricate rectangular micro-grooved array structures on the surface of a mold core. Micro-injection molding is employed to fabricate micro-structured polymers using a micro-structured mold core machined by WEDM. The proposed fabrication method for micro-structured polymer parts may be widely applied and developed due to its low manufacturing cost and possible mass production. The main results are summarized as follows:

1. The regular micro-structures can be accurately processed on the surface of the mold core by WEDM. The chemical compositions of the Ti_3_SiC_2_ mold core surface are hardly changed in the process of electrical discharge machining. The average size machining error of micro-structured mold core using WEDM was 0.394%. The machined surface roughness *R*_a_ at the groove bottom of micro-structured mold core was 1.476 µm.

2. The micro-forming quality of micro-structured polymer is related to the micro-injection molding process parameters. In order to obtain the highest replication rate of 99.12% for micro-formed polymer, the optimal micro-injection molding parameters, namely, jet temperature, melt temperature, injection velocity, holding pressure and holding time are 195 °C, 210 °C, 40 mm/min, 7 Mpa and 5 s, respectively.

3. Compared with injection velocity and jet temperature, the effects of the melt temperature, holding pressure and holding time on the replication rate of micro-structured polymer are more significant. The melt temperature and holding pressure have significant influences on the surface roughness of micro-formed polymer. The minimum surface roughness *R*_a_ at the groove bottom of micro-structured polymer is 0.805 µm.

## Figures and Tables

**Table 1:** The micro-injection molding parameters and corresponding levels in designed experiments.

No.	Jet Temperature *T*_1_ (°C)	Melt Temperature *T*_2_ (°C)	Injection Velocity *V* (mm/s)	Holding Pressure *F* (Mpa)	Holding Time *t* (s)
1	185	210	40	7	5
2	190	210	40	7	5
3	195	210	40	7	5
4	200	210	40	7	5
5	205	210	40	7	5
6	195	200	40	7	5
7	195	205	40	7	5
8	195	215	40	7	5
9	195	220	40	7	5
10	195	210	30	7	5
11	195	210	35	7	5
12	195	210	45	7	5
13	195	210	50	7	5
14	195	210	40	5	5
15	195	210	40	6	5
16	195	210	40	8	5
17	195	210	40	9	5
18	195	210	40	7	3
19	195	210	40	7	4
20	195	210	40	7	6
21	195	210	40	7	7

**Table 2:** The surface roughness and groove size parameters of the machined micro-structured mold core.

Sample	Width *a*_1_ (µm)	Depth *h*_1_ (µm)	Spacing *b*_1_ (µm)	*R*_a_ of Groove Top (µm)	*R*_a_ of Groove Bottom (µm)
Machined mold core	99.683	99.601	100.465	1.354	1.476

**Table 3:** The surface roughness and groove size parameters of micro-structured polymer workpieces.

No.	Width *a*_2_ (µm)	Depth *h*_2_ (µm)	Spacing *b*_2_ (µm)	*R*_a_ of Groove Bottom (µm)	*R*_a_ of Groove Top (µm)
1	100.44	100.967	98.164	0.864	0.996
2	99.069	101.469	98.278	0.871	1.028
3	100.339	101.183	98.503	0.805	0.972
4	98.090	100.667	100.981	0.903	1.166
5	97.087	100.932	100.016	0.943	1.286
6	97.805	100.534	101.451	1.202	1.503
7	98.086	99.998	101.229	1.153	1.396
8	99.414	101.665	98.140	0.874	1.065
9	98.253	100.006	100.986	0.929	1.269
10	100.009	101.48	101.581	0.962	1.287
11	100.05	101.965	98.943	0.804	0.976
12	99.925	101.764	100.834	0.817	0.955
13	98.515	102.109	100.161	0.948	1.241
14	98.198	100.111	101.439	1.163	1.422
15	97.441	99.720	100.570	1.329	1.459
16	98.231	101.845	98.972	1.118	1.326
17	99.185	101.487	101.915	1.267	1.398
18	98.299	101.786	99.534	0.860	1.035
19	98.870	102.251	99.934	0.856	1.016
20	100.909	101.483	100.076	0.822	1.003
21	98.472	99.670	101.289	1.120	1.411
